# A Business Method

**Published:** 1904-03

**Authors:** 


A BUSINESS METHOD.

Just now the business side of dental practice is claiming quite a little attention. All who tackle the subject start with the aphorism that as a rule professional men are bad business men. True, but not this one: He is an "aside" as to location, but has just notions of what is right and wrong; that what is sauce for the goose is also good sauce for the gander. He figures his time worth two dollars an hour. If the patient is half an hour late he must pay for the half hour before the doctor deigns to begin work. It must be *paid* then and there; that's for time, not dentistry. That part settled, he goes on with the work for which the patient specially engaged him. Conversely, if by any chance the doctor is behind, say a quarter of an hour, he hands the patient half a dollar before beginning the service. He has done this from the first, and to-day his methods is so well understood that the patient belated knows just what is expected of him and just what he must do in order to be waited upon. He knows, too, that if the doctor is delayed in beginning his part, there will be a settlement for time forthwith. We haven't the honor of personal acquaintance with the gentleman, but apples to doughnuts he has a bank account and all the credit he may have occasion to use.—*Dental Office and Laboratory.*

Dentists' Fees.—The idea seems to prevail with some that just in proportion as our increased facilities enable us to do more work, they must cut prices, and by so doing make the profession in the eyes of the people a commercial institution. There would be just as much sense in a surgeon, because he has an improved instrument whereby he could perform an operation quicker than usual, cutting his price, or a physician his regular established fee because he is fortunate enough to have a speedy horse or an automobile whereby he can see more patients in a given length of time.—*Dr. J. A. Pearson, Dental Summary.*

